# The potential of aryl hydrocarbon receptor as receptors for metabolic changes in tumors

**DOI:** 10.3389/fonc.2024.1328606

**Published:** 2024-02-16

**Authors:** Zhiying Wang, Yuanqi Zhang, Zhihong Liao, Mingzhang Huang, Xiaorong Shui

**Affiliations:** ^1^ Laboratory of Vascular Surgery, Affiliated Hospital of Guangdong Medical University, Zhanjiang, Guangdong, China; ^2^ Department of Breast Surgery, Affiliated Hospital of Guangdong Medical University, Zhanjiang, Guangdong, China

**Keywords:** aryl hydrocarbon receptor, cancer, metabolic reprogramming, metabolism, tumor microenvironment

## Abstract

Cancer cells can alter their metabolism to meet energy and molecular requirements due to unfavorable environments with oxygen and nutritional deficiencies. Therefore, metabolic reprogramming is common in a tumor microenvironment (TME). Aryl hydrocarbon receptor (AhR) is a ligand-activated nuclear transcription factor, which can be activated by many exogenous and endogenous ligands. Multiple AhR ligands can be produced by both TME and tumor cells. By attaching to various ligands, AhR regulates cancer metabolic reprogramming by dysregulating various metabolic pathways, including glycolysis, lipid metabolism, and nucleotide metabolism. These regulated pathways greatly contribute to cancer cell growth, metastasis, and evading cancer therapies; however, the underlying mechanisms remain unclear. Herein, we review the relationship between TME and metabolism and describe the important role of AhR in cancer regulation. We also focus on recent findings to discuss the idea that AhR acts as a receptor for metabolic changes in tumors, which may provide new perspectives on the direction of AhR research in tumor metabolic reprogramming and future therapeutic interventions.

## 1 Introduction

Cancer is the leading cause of death worldwide, an important barrier to life expectancy, and a cause of considerable economic burden on patients (1). Cancer is caused due to oncogene activation and the loss of cancer suppressors, which causes metabolic reprogramming to provide metabolites and energy needed by the cancer cells to sustain tumorigenesis and survival (2). Under normoxic conditions, cells obtain energy via glycolysis and mitochondrial oxidative phosphorylation, whereas under hypoxic conditions, with compromised mitochondrial function, cells mainly rely on glycolysis (3). The infinite proliferation of cancer cells necessitates urgent requirements for more energy, resulting in an imbalance between the production and consumption of energy and oxygen and a hypoxic environment, followed by a series of dysregulations in various metabolic pathways (4).

Recent studies have revealed considerable differences between the metabolic profiles of cancer and normal tissues, with cancer cells exhibiting varying degrees of alteration in metabolic pathways, including glycolysis, the tricarboxylic acid (TCA) cycle, amino acid, nucleotide, and lipid metabolism. Tumor cells undergo adaptive changes in their metabolic characteristics, a process known as metabolic reprogramming, in response to several interacting factors, including a harsh tumor microenvironment (TME) caused by fast tumor cell proliferation (5). In addition to markedly altered glucose metabolism, tumor cells differ greatly from normal cells regarding nucleotide production and utilization (6); for example, among the three breast cancer subtypes, triple-negative breast cancer (TNBC) has shown the most robust nucleotide biosynthesis compared with that shown by normal breast tissue (7, 8). These metabolic changes and various reactions depend not only on the cancer subtype but also on how the cancer cells interact with the intricate surrounding milieu (9). A particular heterogeneity of cancer cells may result from the changes in cellular metabolic pathways, together with the molecules produced and inefficient oxygen supply, forming the unfavorable TME, which in turn regulates the proliferation and invasion of cancer cells (10). In summary, tumor metabolic reprogramming is a notable contributor to a distinctive TME.

Reportedly, in glioma cells, aryl hydrocarbon receptor (AhR) is activated by kynurenine (Kyn), which is generated by indoleamine 2,3-dioxygenase (IDO), causing tumor-associated macrophage (TAM) accumulation in the TME. Mounting evidence suggests that Kyn-triggered AhR helps tumor cells avoid the immune system (11, 12). In patients with melanoma, high levels of IDO-1 and Kyn have been linked to immunosuppression (13). Although the precise measurement of Kyn in cutaneous melanoma is unknown, it is estimated to be >50 µM in the TME and approximately 2–8 µM in the plasma (depending on the disease type) (14–18). Furthermore, excess reactive oxygen species (ROS) from the TME may trigger the synthesis of antioxidant proteins by activating AhR, enabling tumor cell response to the TME (19). In summary, AhR is an important target for enhancing tumor adaptation, tumor immune evasion, and monitoring changes in the TME.

AhR is a ligand-activated transcription factor that is most commonly associated with xenobiotic ligand metabolism (20). Previous research has discovered that AhR has a crucial role in lipid metabolism, nucleotide *de novo* synthesis, and tumor glycolysis (21–23). AhR ligands include environmentally toxic chemicals, such as 2,3,7,8-tetrachlorodibenzo-p-dioxin (dioxin), and exogenous and endogenous ligands, such as 2-(1’H-indole-3’-carbonyl)-thiazole-4-carboxylic acid methyl ester (ITE) and various tryptophan metabolites (24, 25). Previous studies have shown that AhR ligands may exhibit agonistic or antagonistic activities (26–28). AhR can cause metabolic modifications in cancer cells via the regulation of glycolysis and lipid metabolism by interacting with different ligands (29). However, the causal relationship between AhR and metabolic reprogramming in cancer remains unclear.

ROS are inevitable byproducts of intracellular metabolism (30) and are generated due to active mitochondrial metabolism (31). ROS mediate oxidative stress; however, the true cause is not the formation of ROS but rather the imbalance in space and time between ROS production and detoxification (32). Oncogene activation, upregulation of the phosphoinositide 3-kinase signaling pathway, and hypoxia cause mitochondria in the cancer cells to produce ROS at a higher rate (33–35), producing a harsh TME. Low ROS concentrations, particularly that of H_2_O_2_, favorably control cellular growth and adaptation to metabolic stress (36). Thus, antioxidants are beneficial for tumor cell proliferation and can be targeted to inhibit antioxidants to prevent cancer cell proliferation, tumorigenesis, and metastasis (37). Uric acid (UA), a byproduct of purine metabolism, exhibits antioxidant properties (38) that are likely to have prognostic implications for patients with cancer. Reportedly, AhR can mediate ROS generation (39–41). Interestingly, increased ROS levels in the TME can trigger the production of antioxidant proteins by activating AhR, thereby shielding breast cancer cells from oxidative stress (19). Cancer cells that undergo metabolic reprogramming inevitably produce excess ROS, resulting in a harsh TME. AhR detects ROS and mediates their generation. Hence, AhR, TME, metabolic reprogramming, and ROS are intertwined and maintain a dynamic equilibrium.

Presently, many studies have shown that AhR is involved in the process of cancer metabolic reprogramming by binding with different ligands and that novel interventions targeting AhR may have notable clinical value. In this review, we highlight AhR signaling pathways in the TME and their contributions to tumor survival and invasion. Additionally, we describe the important role of AhR in the regulation of cancer cell metabolism and relevant pathways, which may be used to better understand the potential of anticancer therapies.

## 2 Clinical applications of metabolic reprogramming in tumors

The TME, a crucial location for cancer cell metabolism, is critical for AhR to regulate tumor metabolic reprogramming. Many studies have focused on the relationship between tumor cells and their microenvironments. TME is composed of both cancerous and non-cancerous cells that control tumor growth, progression, and resistance to cancer treatment (42). TME includes extracellular matrix, endothelial cells, cancer-associated fibroblasts, adipocytes, and TAMs. It has been demonstrated that the chemical carcinogen 3-methylcholanthrene activates AhR in CAF, which accelerates the development of breast cancer (43). Kyn-activated AhR produced by CAFs isolated from tumors is associated with tumor drug resistance, and it has been suggested that targeted inhibition of AhR may be a new strategy for the treatment of malignant tumors (44, 45). There is evidence that CAF is associated with tumor metabolism (46), and interestingly, AhR can be activated by small molecules produced in metabolism (47). In glioblastoma, kyn activates AhR in TAM to regulate its function and T-cell immunity, correlating with poor tumor prognosis (11). Tryptophan-derived metabolites can aid immune escape from tumors by activating the AhR of TAM, and expression of the AhR in TAM has a profound impact on tumor growth and TME (48). In TME, the expression of AhR in CAF and TAM is closely related to metabolism and tumor progression.

TME studies have mainly focused on metabolic reprogramming, which is considered one of the key factors promoting cancer development (49). The dysregulation of tumor cell metabolism results in hypoxia, an acidic TME, and the depletion of sensitive metabolites in the cells (50), which enables the invasion of effector T cells to compete with the tumor for metabolites and compromise their function (51, 52). Cellular carcinogenesis causes metabolic pathways to be dysregulated, and these altered metabolic pathways, in turn, provide cancer cells with a better chance of survival under hypoxic environments and confer them the ability to proliferate and survive at a high rate. Reportedly, metabolic byproducts of cancer cells can regulate the function of tumor-infiltrating immune cells and offer numerous benefits. For example, lactic acid released by cancer cells through glycolysis promotes the polarization of immune cells toward an immunosuppressive phenotype (53, 54). Changes in the metabolism of cancer cells also exert a considerable effect on other TME components, such as non-cancerous cells, in the microenvironment, which can stimulate the migration of cancer cells and mediate pro-cancerous activities (9). Because cancer cells grow much faster than normal cells because of metabolic reprogramming (55), metabolic reprogramming and the TME have become popular research topics. Studies on tumor heterogeneity have focused on the development of immunosuppression by tumor cells in response to glucose competition with normal tissues and the increased release of lactate from the microenvironment following metabolism (56, 57). Tumor metabolism is controllable, and clinical therapy that reprograms the TME greatly affects the chemotherapy, radiation, and targeted therapy on tumor cells. Through the use of nano-delivery technologies, the T cell activator anti-CD28-coupled aryl hydrocarbon receptor (AhR) inhibitor (CH223191) can be encapsulated to modify the tumor immune milieu and successfully prevent tumor cell metastasis (58).

There have been some successful attempts at reprogramming tumor metabolism worldwide. A meta-analysis showed that the overexpression of glucose transporter protein 1 (GLUT1) in solid cancers is linked to the poor prognosis of many tumor types, suggesting that direct GLUT1 targeting could be a promising treatment strategy for solid cancers (59). Serine biosynthesis is frequently increased in various cancer cells, and overexpressed genes are involved in nucleotide synthesis, antioxidant defense, and methylation responses in breast cancer cells (60, 61). Fatty acid synthase (FASN) is an oncogene and is involved in cancer-associated metabolic reprogramming, and FASN-targeted drugs are in clinical development and trials (62). In addition to recent studies on glucose, amino acid, and lipid metabolisms, the study of nucleotide metabolism and its metabolites in the TME metabolic reprogramming has been a hot spot. Recent research demonstrated that purine metabolism in the TME causes heterogeneity in macrophages (63), and that proline isomerase of CAF in TME targets pancreatic ductal adenocarcinoma synergetically with gemcitabine, the antipyrimidine metabolizing medication, to facilitate the elimination of the tumor by immunochemotherapy (64). It’s interesting to note that changes in AhR expression are correspond to susceptibility and resistance to the clinical antimetabolic chemotherapy medicines gemcitabine and cytarabine, which are currently widely utilized (65, 66).

**Figure 1:**
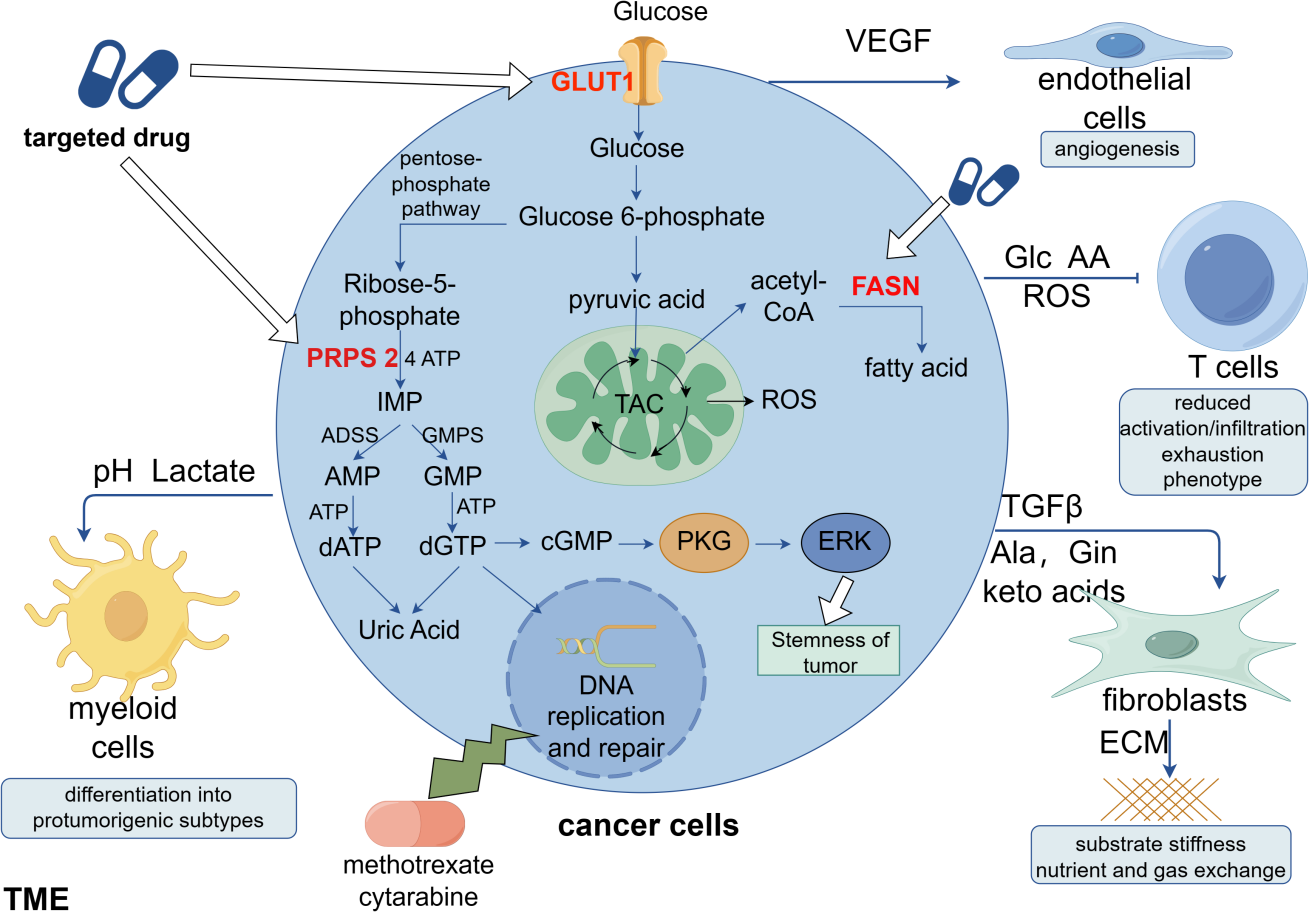
Clinical applications of metabolic reprogramming in tumors. Cancer cells can secrete a variety of factors to stimulate non-cancer cells in the TME, favoring their own proliferation, migration and immune escape. Studies indicate that new targets for anti-tumor metabolism are likely to include phosphoribosyl pyrophosphate synthase 2 (PRPS 2), glucose transporter protein 1 (GLUT1), and fatty acid synthase (FASN). Among these, it has been demonstrated that the nucleotide *de novo* synthesis-related enzyme PRPS 2 is linked to the stemness of breast cancer, and its efficient suppression will prevent lung metastases of breast cancer. Targeting nucleotide *de novo* synthesis has significant promise since, in contrast to cancer cells, normal cells obtain nucleotides through remedial synthesis. This is because ab initio nucleotide synthesis consumes a considerable quantity of ATP.

The synthesis of nucleotides and deoxyribonucleotides is the first metabolic route to be extensively studied and successfully targeted in cancer therapy (67). Deoxyribonucleoside triphosphates (dNTPs), which are necessary for DNA replication and transmission of the entire genome to the next generation, are required by all dividing cells during the S phase of the cell cycle. Additionally, compared with normal cells, tumor cells that are highly replicating and proliferating have more active nucleotide metabolism. Targeting DNA replication is another early cancer therapeutic strategy (68). Early identified antimetabolic chemotherapeutic agents include folate antagonists, pyrimidines, and purines such as methotrexate, cytarabine, and fluorouracil. However, these interfere with DNA replication and synthesis and greatly damage autologous cells, searching for specific targets is an urgent problem. Both remedial and *de novo* synthesis can provide nucleotides for cellular needs, and remedial synthesis serves as the main route for nucleotide acquisition in healthy cells. In a recent study, breast cancer cells undergoing lung metastasis were found to have considerably higher levels of phosphoribosyl pyrophosphate synthase 2 (PRPS2), which is a crucial gene for *de novo* nucleotide synthesis. This gene leads to the production of more cyclic guanosine monophosphate (cGMP), which in turn activates the cGMP-dependent protein kinase G and downstream mitogen-activated protein kinase (MAPK) pathways, thereby increasing tumor stemness. Tumor stemness can be considerably reduced and lung metastasis can be prevented in breast cancer cells by silencing the PRPS2 gene to block *de novo* nucleotide synthesis. The metabolic signature of metastatic breast cancer cells is accelerated by nucleotide synthesis from scratch, and its metabolites can modify signaling pathways to support breast cancer stemness and metastasis (69) (**Figure 1**).

Nucleotide biosynthesis requires a large amount of energy, as shown by the purine synthesis pathway, which requires five adenosine triphosphate molecules, two glutamine and formic acid molecules, and one glycine, aspartic acid, and carbon dioxide molecule to produce one hypoxanthine nucleotide molecule (70). Metabolic reprogramming is crucial in the fight against tumors because the energy metabolism of malignant cells differs greatly from that of normal cells. Many studies have revealed that AhR regulates metabolic pathways and ROS of tumors to varying degrees, offering notable potential for antitumor research (19, 21, 23, 40, 41, 71–73). Recent studies have shown that ROS are involved in metabolic regulation by activating the ribotoxic stress response (RSR) in cellular models (74).

## 3 Role of AhR in tumor progression

AhR functions as a complex metabolic regulator and transcription factor in most cancer cells. AhR is a ligand-activated receptor for aryl hydrocarbons. When activated from its dormant state in the cytoplasm, AhR translocates to the nucleus and activates the transcription of its target genes (75). In tumor immune escape, AhR plays a key role in the immunosuppressive phenotype of the TME in cancer cells and is closely related to human amino acid metabolism as a sensor of tryptophan metabolites and is also a powerful immune system regulator (48). AhR activation enhances tumor aggressiveness, reduces cluster of differentiation (CD)8 T cell (76) and macrophage (48) antitumor immunity, and aids tumor cells in evading immune responses (77). In summary, AhR overexpression helps tumor cells evade the immune system by sending inhibitory signals to the immune cells through the TME.

In addition to its role in tumor immune evasion, AhR may also play a crucial role in the growth of tumor cells by controlling the cell cycle. In liver cancer, AhR activation prevents tumor cells from entering the G0/G1 phase, which reduces DNA replication and prevents cell proliferation. High levels of tetraploidy have been associated with an increased risk of tumor formation (78), and the existing evidence shows that tumors typically contain chromosomes close to the tetraploid, and the uncontrolled proliferation of tetraploid cells trigger tumor formation (79). Sustained DNA damage has been observed in AhR-deficient hepatocytes, which is detrimental to the entry of cells into the tetraploid phase of proliferation. AhR, as a tumor suppressor, can be activated by controlling the expression of inflammatory cytokines, DNA damage, and cell proliferation (78). In a study, AhR mutant mice showed increased development of liver tumors, and AhR agonists suppressed cholesterol regulatory element-binding protein (SREBP)2 and stopped tumor progression in mice (28). Studies on hepatocellular carcinoma have shown that the activation of AhR can have an oncogenic effect, as opposed to immune escape. Thus, it is evident that the regulatory activities of AhR play intricate roles in tumors and are closely linked to tryptophan metabolism (80).

It is worth investigating whether AhR activation promotes or hinders tumor formation, and studies on breast cancer have readily demonstrated this paradox. When AhR was knocked down by short interfering RNA in two different types of breast cancer cells, BT474 and MDA-MB-468, its regulatory effect on cell proliferation was enhanced in BT474 cells, whereas no effects were detected on the proliferation of MDA-MB-468 cells (81). Animal and cellular studies on the breast cancer cell line MCF-7 have shown that AhR expression is not necessary for mammary carcinogenesis (82) and that the MCF-7 cell proliferation is unaffected by AhR deficiency (83). In contrast, AhR overexpression promotes MCF-7 cell proliferation (84). Furthermore, whether AhR promotes or inhibits malignancy in TNBC MDA-MB-231 cells remains debatable (85–87). According to previous studies, ligands for AhR can either be exogenous or endogenous, and they can function in an agonistic or antagonistic manner (88). Thus, AhR expression alone plays various roles in breast cancer depending on the ligand to which it binds and its effects on different cell types. This suggests that AhR may serve as a relay for pro- or oncogenic factors, and its binding to various AhR ligands helps control the growth and migration of breast cancer cells. Protein kinase A complexes of AhR and nuclear factor-κB (NF-κB) RelB can attach to chemokine-specific binding sites to activate inflammatory factors (89). This promotes the proliferation and migration of breast cancer cells. Additionally, AhR can interact with NF-κB RelA and lead to an increase in cellular-master regulator of cell cycle entry and proliferative metabolism (Myc) levels in MCF-7 cells, which further triggers carcinogenesis (90).

Epithelial-mesenchymal transition (EMT) is the process that epithelial cells lose their polarized shape and acquire the capacity of migration and invasion. It occurs in the early stages of tumor metastasis and is considered to be an important cause of cancer metastasis (91). EMT biomarkers such as waveform protein, N-calmodulin, and MMP 9 are overexpressed in cancer and involved in the promotion of cancer metastasis (92). Many studies have shown that AhR activity leads to loss of cell contact inhibition and alterations in extracellular matrix remodeling, and it is also found that AhR plays critical role in EMT induction (93–95). According to Dai et al., kyn stimulates AhR in renal cell carcinoma to promote infiltration, migration, and EMT progression (96). MMP, a biomarker of EMT, is a family of zinc-dependent endoproteases which could degrade the extracellular matrix to promote cell proliferation and migration (97). MMP could also affect the TME of malignant tumors and support EMT by inducing invasive and metastatic tendencies of cancer cells (98). In esophageal cancer cells, knockdown of AhR gene inhibits tumor progression by down-regulating the expression levels of MMP 1, MMP 2, and MMP 9 (99). Different thyroid carcinoma cell types are promoted to express MMP1, MMP2, and MMP9 differently by kyn-activated AhR (95). TCDD-activated AhR upregulates MMP9 expression activity in a variety of malignant tumors (100–104). As a result, the activation of AhR in different tumors demonstrated a consistent promotion of MMPs, which therefore affect EMT and the further tumor progression.

In addition to MMP, other markers of EMT are also widely studied. TDO 2 regulates metastasis and invasion of hepatocellular carcinoma by activating the Kyn-AhR pathway to promote EMT in cancer cells (104). In thyroid carcinogenesis cells, kyn activates AhR expression, then promotes an increase in fibronectin, SLUG, and N-calmodulin and a decrease in E-calmodulin, which resulted in increased cell invasion and motility. The authors also found that AhR has a close correlation with EMT, with AhR being essential in managing the immunosuppressive milieu, it therefore triggers the development of both EMT and an immunosuppressive TME (95). 3,3’-Diindolylmethane could reverse the EMT process by regulating AhR inhibition of the EGFR/RhoA/ROCK 1/NF-κB/COX 2/PGE 2 pathway, it is reported that 3,3’-Diindolylmethane down-regulated the expression of mesenchymal cell markers including β-Catenin, Vimentin, and Slug, and upregulated the epithelial cell marker Claudin-1 (105). TCDD-activated AhR also promotes EMT in ovarian cancer cells (106), and the current study demonstrated that both ligand-activated AhR promote EMT. However, direct overexpression of AhR showed different regulating roles in different cancers. In lung cancer cells, cells overexpressing AhR exhibited lower cell mobility, high expression of E-cadherin and low expression of waveform proteins (biomarkers associated with EMT), suggesting that higher AhR expression are associated with lower cell motility (107). Studies on gastric cancer cells indicated that ROS mediates the KYNU-kyn-AhR signaling pathway to influence EMT capacity (108). Direct overexpression of AhR leads to the opposite effect, however, the role of ligand-activated AhR in the EMT of malignancies is better established. Therefore, AhR has its potential to be the receptor for metabolic changes in tumors.

Although intricate processes are involved, AhR plays a crucial role in tumorigenesis. The activation of the tryptophan 2,3-dioxygenase-2 (TDO2)–Kyn–AhR pathway promoted liver metastases of colon cancer in a mouse model by aiding in immune evasion and maintaining stemness (109). As AhR can bind to various ligands, we can deduce that it plays various roles in malignancies. However, because the diverse roles of AhR impede a thorough investigation of its mechanisms, it is imperative to identify a broad path for future research on the involvement of AhR in carcinogenesis and development.

## 4 Effect of different ligand-activated AhR on tumor glucose metabolism

Cancer cells support their metabolism through glycolysis, both aerobic glycolysis (also called the Warburg effect) and anaerobic glycolysis, to provide biosynthetic molecules and energy for the survival and development of cancer cells (55); therefore, robust glycolysis is considered a hallmark of cancer metabolism. However, the effects of AhR on augmented glycolysis remain unclear. Most cells receive sufficient energy to maintain cellular activity through efficient oxidative phosphorylation of glucose in normoxic environments; however, in hypoxic environments, glucose can only be used to produce energy through inefficient anaerobic glycolysis (110, 111). Interestingly, increased glycolytic metabolism can indicate that cancer cells are actively reproducing because glycolysis serves as the primary energy source for cancer cells under both normoxic and hypoxic conditions (55). Numerous studies have suggested that AhR may be an important regulator of glycolytic gene expression and glycolytic endpoints (21, 112–116).

Reportedly, TDO2 enhances the Kyn pathway (KP), thereby producing excess Kyn, which further activates AhR and upregulates CXC chemokine ligand 5 (CXCL5). CXCL5 recruits TAM into the TME and promotes TAM polarization and abundance, leading to active glycolysis in cancer cells and thereby promoting cancer cell proliferation and survival. AhR modulates glucose absorption and total glycolytic flux, in addition to upregulating CXCL5 by affecting several glycolytic genes, including GLUT1, hexokinase (HK) 1/2, and phosphofructokinase-liver type (23). The activation of the cytokine/IDO/Kyn/AhR pathway in pancreatic cancer cells can shield them from inflammation in the hostile TME and facilitate their adaptation to it (117).

**Figure 2:**
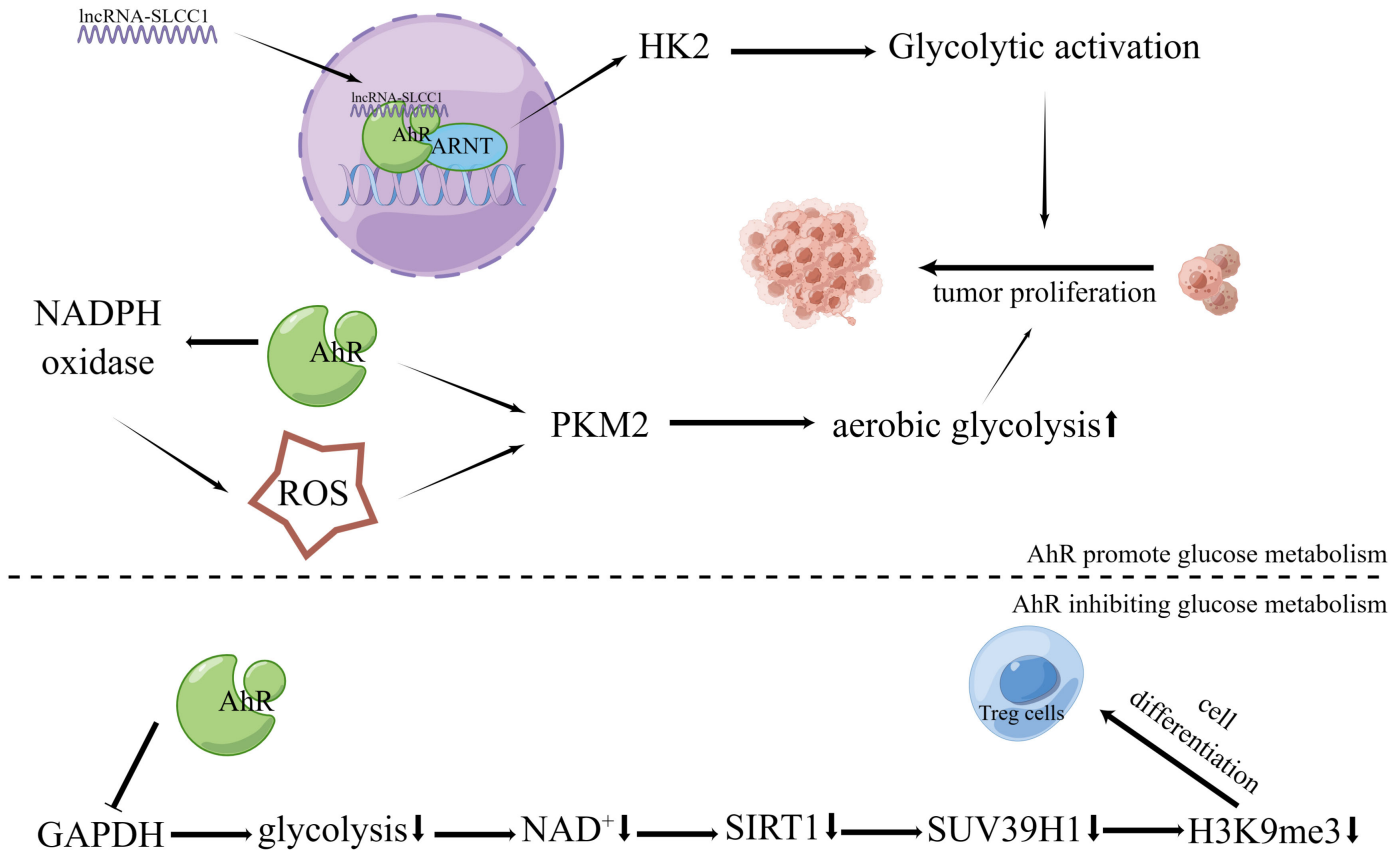
Effects of AhR in tumor energy metabolism. The lncRNA-SLCC1 binds to the AhR and increases the expression of HK2, which activates glycolytic metabolism and promotes tumor growth. As a result of AhR’s activation of NADPH oxidase and subsequent upregulation in ROS production, the expression level of PKM2, the rate-limiting enzyme of aerobic glycolysis, is further increased. This boosts the aerobic glycolysis of tumor cells and encourages the growth of tumor cells via suppressing GAPDH and preventing glycolysis, AhR activation promotes Treg development via controlling the NAD+/SIRT1/SUV39H1/H3K9me3 signaling pathway.

Recently, it was discovered that AhR binds to lncRNA and actively controls the expression of the glycolytic enzyme gene hexokinase 2 (HK2), stimulating glycolytic metabolism and accelerating tumor growth (72) (**Figure 2**). Another study found that HK2 is a transcriptional target of AhR, and over-expression of HK2 could in turn alter AhR gene expression and modulate its activity (118). Additionally, in oncogene MYC-expressing rat fibroblast cells, AhR deletion has been shown to reduce intracellular glucose and pyruvate as well as the expression of enzymes associated to glycolysis and tricarboxylic acid (21). In addition, AhR activated by 2,3’,4,4’,5-pentachlorobiphenyl (PCB118) increased ROS production by up-regulating nicotinamide adenine dinucleotide phosphate (NADPH) oxidase. In the last stage of the glycolytic process, ROS can further upregulate the expression level of the rate-limiting enzyme M2-type pyruvate kinase (PKM2) to improve glucose metabolism in tumor cells (71).

Reduced glucose uptake and mitochondrial activity are correlated with downregulated GLUTs due to 2,3,7,8-tetrachlorodibenzo-p-dioxin (TCDD)-activated AhR, which impacts glucose transport and utilization in pluripotent embryonal cancer cells (119). While assays for glycolytic products have revealed a considerable reduction in glycolytic flux, AhR activation has been shown to decrease the rate of glucose uptake and the formation of pyruvate and lactate (115). The glycolytic process considerably decreased in the colon cells after activation of AhR by Norisoboldine, indicating that AhR activation interferes with the glycolytic pathway by suppressing the production of glyceraldehyde-3-phosphate dehydrogenase (GAPDH) (120) (**Figure 2**). Consequently, AhR showed important regulating roles in the control of glucose metabolism by binding with different ligands. In addition to glycolysis, cancer cells harness lipid metabolism to obtain the energy and molecules needed for proliferation, survival, invasion, and adaptation to the TME (121).

## 5 Potential regulatory mechanisms of AhR in tumor lipid metabolism

Lipids are involved in energy metabolism and are important components of the cell membranes and secondary messengers. Because tumors have high metabolic demands and large fatty acids consumption, therefore, cancer cells are different from normal cells in terms of how they absorbing exogenous fatty acids and producing endogenous fatty acids. Specialized transporter proteins are necessary for the effective passage of exogenous FA across the plasma membrane. Tumors exhibit a significant increase in the gene and protein expression levels of these fatty acid transporter proteins; of them the most well studied protein is CD 36, which is commonly referred to as fatty acid translocase (FAT) (122, 123). AhR activation was linked to increased CD36 expression, and siAhR efficiently reduced lipoxin A4-induced CD36 overexpression and lipid uptake (124).

In healthy tissues, only hepatocytes and adipocytes are capable of *de novo* lipogenesis; however, cancer cells also have the ability to reactivate this anabolic process (125). Key regulators of adipogenesis, including sterol regulatory element binding protein (SREBP), acetyl coenzyme A carboxylase (ACC), FASN and stearoyl coenzyme A desaturase 1 (SCD-1), are detected to be significantly up-regulated in various human cancers (125–128). According to further analysis, we found that these enzymes connected to *de novo* adipogenesis can be effectively regulated by AhR. The expression level of SCD-1 can be attenuated by inhibiting the expression of AhR (129). Indole-3-acetic acid (IAA)-activated AhR negatively regulates SREBP-1c and FASN (130). In colon tumor cells, inhibition of AhR activity decreases the expression levels of SCD-1, a key enzyme of the biosynthetic pathway, and SREBP, a transcriptional regulator of adipogenesis, to restrict cancer cell proliferation in a cell-specific manner (73). Although there has been very limit research on AhR’s role in tumor lipid metabolism, it is found that AhR influences many of the genes that are up-regulated in tumors and are linked to lipid metabolism regulation, including CD36, SCD-1, SREBP, and FASN. Therefore, AhR has great potential in regulating tumor lipid metabolism and worth more further investigation.

## 6 AhR, UA, and ROS are jointly involved in the adaptive regulation of TME by tumors

By integrating metabolomics and transcriptomics, it has been discovered that AhR regulates MYC expression to modulate glycolysis and *de novo* pyrimidine production in the cells. Metabolomics data also showed a decrease in uridine monophosphate (UMP), and AhR silencing resulted in decreased expression and translation of genes related to pyrimidine ab initio synthesis (21). No previous study has reported whether purine metabolism in nucleic acids is related to AhR, however, we found that AhR may play important roles in regulating nucleotide metabolism.

UA is a metabolite of purines, and a large number of clinical studies have shown that UA is associated with tumorigenesis and progression and has received wide attention. Breast cancer cell proliferation and migration have been reported to be affected by increased *de novo* nucleotide synthesis, and UA was used to assess the prognosis of patients with breast cancer and as a feedback regulator of signaling pathways (69). Serum hyperuric acid (SUA) has been shown to affect the course of treatment and prognosis of patients with cancer, and prospective studies have reported that SUA increases the probability of cancer-induced mortality (131–134). In contrast, clinical evidence from some regions suggests that UA exhibits an anticancer effect due to its antioxidant activity (135–138), and fundamental research revealed that UA exerts anticancer effects by promoting dendritic cell maturation, which thereafter triggers an immune rejection response against tumors (139). Reportedly, UA exerts antioxidant effects in extracellular environments but exerts pro-oxidant effects in intracellular environments. UA may function as an antioxidant, scavenge oxygen free radicals, and reduce the production of carcinogenic ROS, which increases the mutation rate of cells, thereby increasing the risk of carcinogenesis, indicating that targeting UA may lower the risk of cancer (38, 140). However, by functioning as a pro-oxidant, UA may penetrate normal cells and contribute to cancer progression by enhancing tumor cell proliferation, migration, and survival through ROS and inflammatory stress (141).

**Figure 3:**
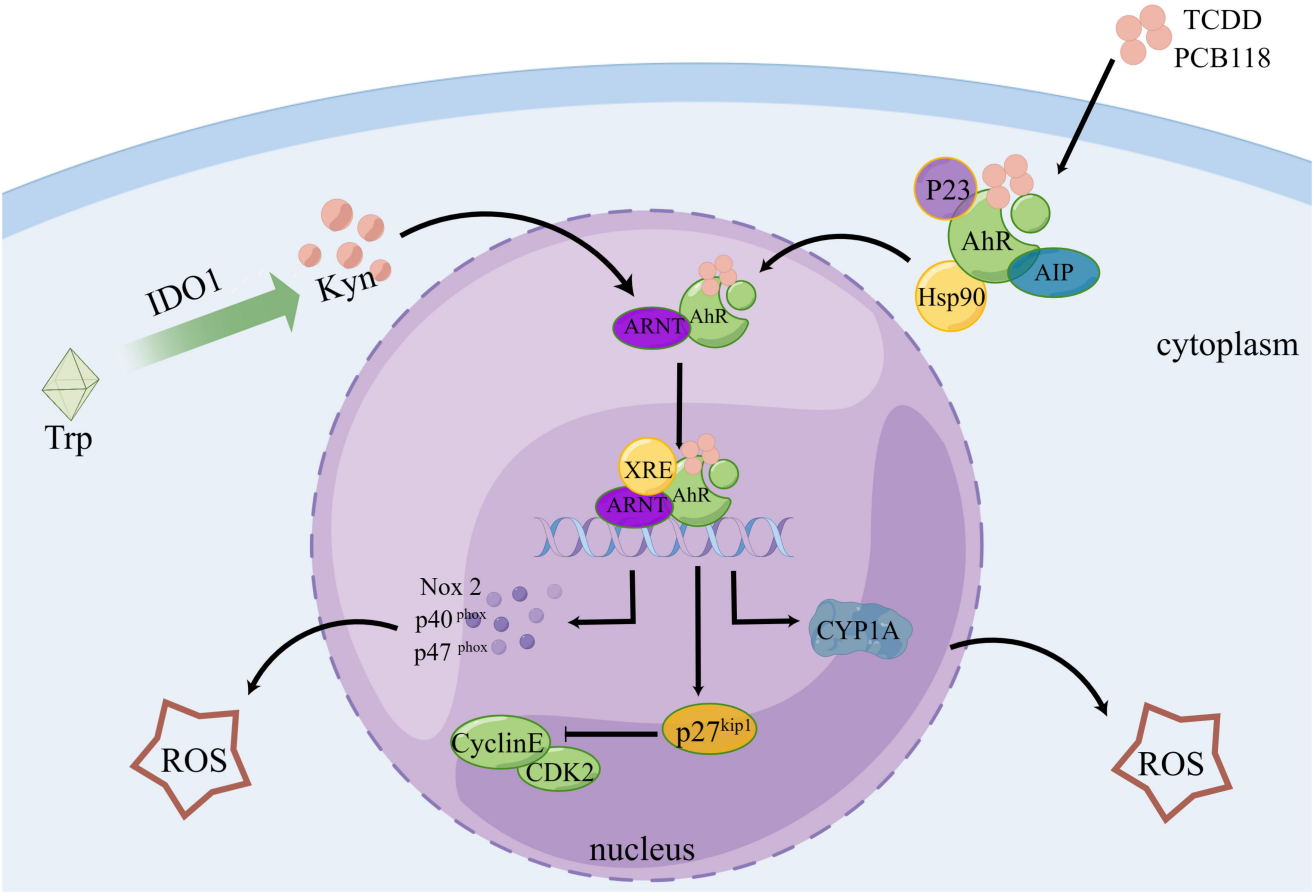
AhR controls the cell cycle and produces ROS. Following TCDD and PCB118 activation of the AhR complex with P23, AIP, Hsp90, etc., AhR is transported to the nucleus where it binds to ARNT to form a heterodimer. This heterodimer then binds to XRE to activate the expression of XRE-controlled genes like CYP1A, Nox2, P27Kip1, etc. ROS are regulated by CYP1A, Nox2, P40, P47, and other proteins, and P27Kip1 can block the cell cycle. Additionally, Kyn, a byproduct of IDO1-controlled tryptophan metabolism, plays a role in AhR regulation.

According to certain theories, AhR can regulate the transcription of some CYPs, which can mediate AhR to generate ROS, and AhR is crucial for the burst of ROS that occurs following reoxygenation (142). After activation, AhR translocates from its inhibitor proteins to the nucleus and actively forms heterodimers with ARNT (143, 144). The transcription of genes, including CYP1A, NADPH oxidase 2, P40phox, and P47phox, is stimulated by the binding of these complex proteins to dioxin- or xenobiotic-responsive elements, which is a key step in the development of oxidative stress-mediated breast cancer (145). An imbalance between ROS production and antioxidant scavenging activity induces oxidative stress (146, 147). Additionally, it has been reported that pentachlorobiphenyl stimulates NADPH oxidase via AhR, thereby increasing ROS generation (71). Cell cycle protein-dependent kinase inhibitor 1 B (p27kip1) coordinates cell cycle progression by inhibiting cyclin-dependent kinase complexes (148). AhR was established as a direct regulator of p27Kip1 transcription. AhR activation in hepatocellular carcinoma upregulates p27kip1, which suppresses hepatocellular carcinoma cell proliferation (149) (**Figure 3**). Recently, it has been found that ROS can activate RSR signaling to participate in the metabolic regulation of cells (74).

**Figure 4:**
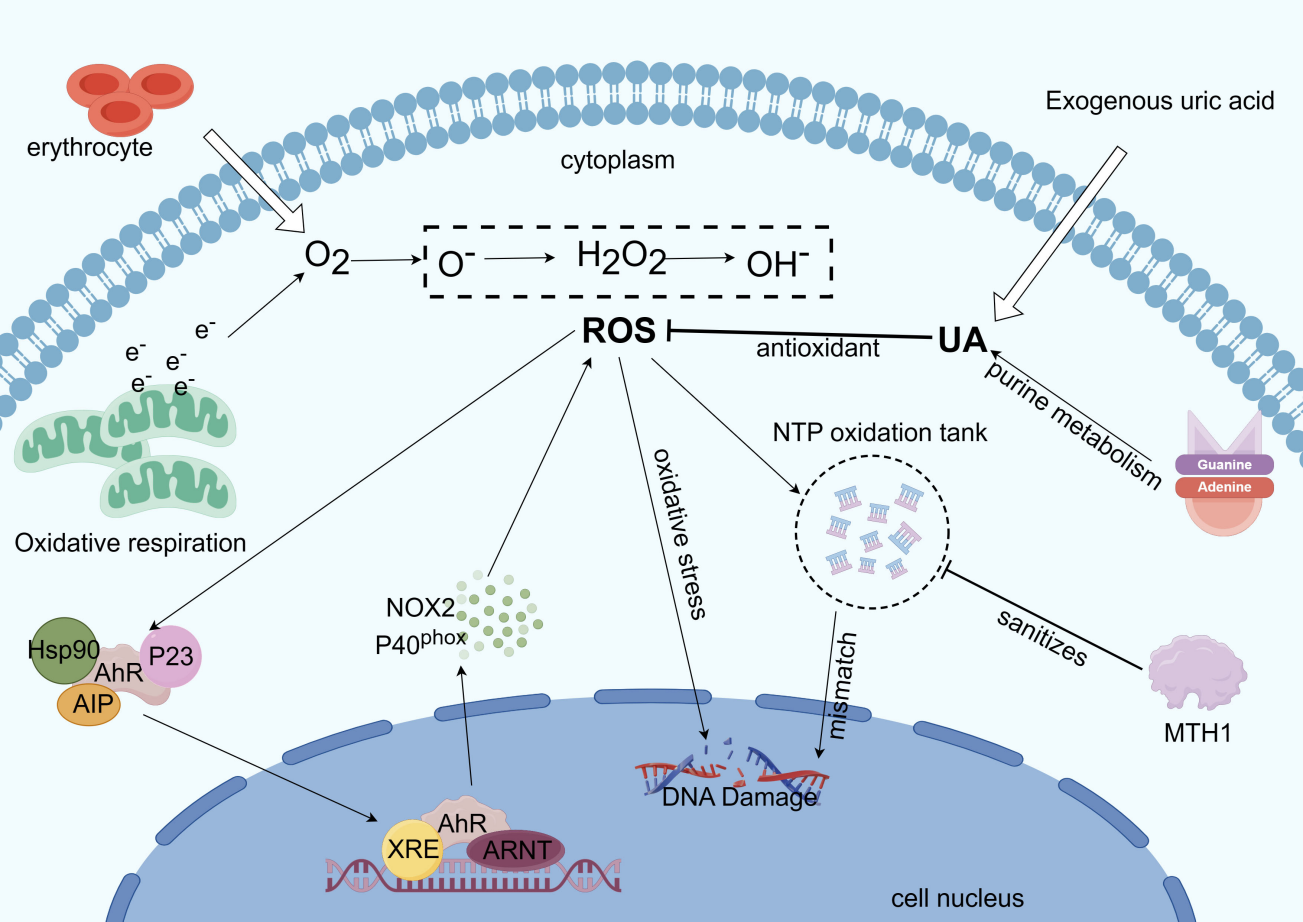
Mechanism of AhR involvement in intracellular ROS regulation. Excess ROS is produced by mitochondrial metabolism when electrons are overworked (32). ROS also form NTP oxidation pools, dope oxidized dNTPs, and cause oxidative stress, which damages DNA during tumor cell replication. ROS can also trigger the transcription of genes like CYPs and NOX2 to react with the ROS (19). Reactive oxygen species generation is suppressed by UA’s antioxidant action. Tumor cell growth benefits from base mismatch reduction, mutation reduction, and purification of the NTP oxidation pool—all of which are achieved by MTH1 (153).

Indirect oxidative damage to DNA and free radicals in the cellular and mitochondrial dNTP pools can result from redox regulation dysfunction and increased ROS levels (150), and the incorporation of these oxidized nucleotides into DNA synthesis can result in mismatches, mutations, and cell death (151, 152). Eukaryotic cells have two functionally distinct dNTP pools. The smaller pool is used for mitochondrial DNA replication and is available throughout the cell cycle, whereas the other pool is used for genomic DNA replication and repair in the nucleus and is available primarily during the S phase (67). The human MutT homolog 1 (MTH1) protein is required for the effective survival of cancer cells, but not for normal noncancerous cells. When oxidized dNTPs are present in cancer cells due to oxidative stress and excessive ROS under harsh conditions, MTH1 overexpression prevents DNA damage induced by oxidized nucleotides during replication. Owing to the strict redox control in normal cells than in cancer cells, normal cells may appear less dependent on MTH 1 activity (153). In summary, MTH1 activity facilitates the alleviation of DNA damage in tumor cells via AhR-mediated ROS production (**Figure 4**).

## 7 Conclusion and perspective

In conclusion, AhR is crucial in the regulation of cellular metabolism, especially in tumor metabolic reprogramming. Many study has been focus on glucose metabolism, but no unified conclusions are achieved on whether AhR upregulation promotes or inhibits glucose metabolism (23, 72). In terms of lipid metabolism, the involvement of AhR in tumor lipid metabolism has been reported only in colon cancer (73). However, we found that AhR pays significant roles in the regulation of genes related to lipid metabolism changes in a variety of cancer cells. These findings highlight the pivotal role of AhR in metabolic control. Unfortunately, no great advancement has been made in research on nucleotide metabolism in initial clinical tumor therapy trials targeting AhR.

Many studies have revealed that AhR may control ROS in the TME, creating an environment favorable for tumor growth and migration, and even affecting cancer treatment (41). From the above, we can draw various conclusions. The regulatory function of AhR in cell glucose, lipid, and nucleotide metabolism is undeniably present and can aid tumor immune escape by modifying immune cells in the TME. While tumor metabolic reprogramming helps cancer cells proliferate quickly, it also unavoidably produces a harsh TME, as indicated by increased ROS levels. Although excess ROS accumulation is harmful to cell proliferation, AhR can detect excess ROS and trigger the production of antioxidant proteins, thereby shielding cancer cells from oxidative stress. The effects of ROS on tumor cells can also be mitigated by MTH1-mediated cleansing of oxidized dNTPs (153). UA, the final byproduct of human purine metabolism, exhibits antioxidant properties (38) and reduces intracellular ROS, which in turn reduces AhR activation and downregulates P27kip1, promoting tumor cell cycle and progression (**Figure 4**). In conclusion, AhR has great potential to regulate tumor metabolic reprogramming by sensing changes in the TME.

## Author contributions

ZW: Writing – original draft. YZ: Funding acquisition, Writing – review & editing. ZL: Writing – review & editing. MH: Writing – review & editing. XS: Funding acquisition, Supervision, Writing – review & editing.
